# Orally administered endoxifen inhibits tumor growth in melanoma-bearing mice

**DOI:** 10.1186/s11658-017-0068-7

**Published:** 2018-01-03

**Authors:** Paul Chen, Saifuddin Sheikh, Ateeq Ahmad, Shoukath M. Ali, Moghis U. Ahmad, Imran Ahmad

**Affiliations:** Jina Pharmaceuticals, Inc., 28100 N. Ashley Circle, Suite 103, Libertyville, IL 60048 USA

**Keywords:** Endoxifen, Melanoma tumor model, Tamoxifen, Safety, Efficacy

## Abstract

Endoxifen, an active metabolite of tamoxifen, has been shown to be an effective anti-estrogenic agent in estrogen receptor-positive breast cancer patients. In melanoma, estrogen receptor expression is shown to be associated with disease progression. However, the therapeutic benefit of endoxifen in melanoma has not yet been evaluated. Here, we present the first demonstration of the anti-melanogenic activity of endoxifen in vitro and in vivo.

The in vitro cytotoxic effect of endoxifen was tested using a cell viability assay. The in vivo anti-melanogenic activity was evaluated in B16F10 cell-bearing C57BL/6 mice, a mouse melanoma model. The general toxicity was tested in Swiss albino mice.

Endoxifen exhibited greater activity against melanoma cell lines. Treatment of B16F10 mouse and SK-MEL-5 human melanoma cell lines with 10 μM of endoxifen for 48 h respectively resulted in 93.6 and 92.5% cell death. Orally administered endoxifen, at dose levels of 4 and 8 mg/kg body weight/day for 20 consecutive days, respectively reduced metastatic melanoma nodules in the lungs by 26.7 and 82.7%.

Endoxifen was found to be a safe and effective anti-melanogenic agent in animal studies.

## Introduction

The presence of estrogen receptor (ER) in melanoma cells prompted research on the potential to use selective estrogen receptor modulators such as tamoxifen to treat patients with melanoma [1, 2]. Early clinical studies showed some clinical benefit, especially when used in combination with other therapeutics [3]. However, additional results from randomized and controlled clinical studies were disappointing [4, 5].

With the growth in understanding of the importance of cytochrome P450 2D6 (CYP2D6) polymorphism and its relationship with the therapeutic outcome of tamoxifen in patients with breast cancer [6], we can now partly attribute the unpredictable results from early melanoma studies to CYP2D6 polymorphism [7]. Drug–drug interactions have also been shown to affect tamoxifen activity. The effectiveness of endoxifen, an active metabolite of tamoxifen, is independent of CYP2D6, and its unique therapeutic benefit was recently confirmed in patients with breast cancer [8, 9].

In this study, the anti-melanogenic activity of endoxifen was tested in human and mouse melanoma cell lines. The in vivo efficacy and toxicity profiles of endoxifen were respectively evaluated in a melanoma model in C57BL/6 mice and Swiss albino mice. Here, we report for the first time on the anti-melanogenic efficacy of endoxifen in a mouse melanoma model.

## Materials and methods

### Drugs and chemicals

Tamoxifen was purchased from Toronto Research Chemicals. Endoxifen (99.8% Z-endoxifen) was obtained from Intas Pharmaceuticals Ltd. Ethyl alcohol (200 proof) was from Sigma-Aldrich. The WST-1 (4-[3–4-iodophenyl]-2-(4-nitrophenyl)-2*H*-5-tetrazolio)-1,3-benzene disulfonate) cell viability kit was purchased from Roche Diagnostic.

### Cell culture

Mouse melanoma cell line B16F10 (ATCC) was maintained in DMEM supplemented with 10% FBS, 2 mM glutamine, 100 units/ml penicillin and 100 μg/ml streptomycin at 37 °C in a humidified atmosphere of 5% CO_2_. All culture media and related reagents were purchased from Thermo Fisher Invitrogen.

### In vitro activity of endoxifen

The effect of endoxifen on B16F10 cells was evaluated using the WST-1 cell viability assay. Approximately 3 × 10^3^ cells were aliquoted in triplicate into a 96-well culture plate and cultured overnight. Endoxifen was diluted with culture medium in an ethyl alcohol stock solution and added to each well at a final concentration of 10 μM. After 48 h incubation, the WST-1 assay was performed according to the manufacturer’s protocol.

The effect of endoxifen in human melanoma cell lines was evaluated using the sulforhodamine B (SRB) assay in the single-dose (10 μM) format from the National Cancer Institute. Briefly, approximately 5 × 10^3^ cells/well were inoculated into a 96-well plate, followed by 24 h incubation prior to addition of endoxifen. After 48 h incubation with endoxifen, the cells were fixed with cold trichloroacetic acid (TCA), washed and stained with 100 μl of 0.4% SRB solution per well for 30 min. The absorbance was read on a plate reader at a wavelength of 515 nm.

### Preclinical safety study in mice

All animal studies were performed according to relevant in-house SOPs under the guidelines of the National Institutes of Health guide for the care and use of laboratory animals (NIH Publications No. 8023, revised 1996).

A subchronic oral toxicity study was performed in Swiss albino mice. Male and female mice (6 per group per dose per sex), of 6 to 8 weeks of age, were given endoxifen citrate daily via oral gavage at dose levels of 0.2, 0.4, 0.8 or 8 mg/kg body weight for 28 days. The control group was only given the vehicle. The mice were monitored for clinical signs, mortality and changes in body weight over the 28 days. On day 29, the blood and organs were collected for hematology, clinical chemistry, gross pathology and histopathology.

### Melanoma model

Female C57/BL6 mice, 5 to 6 weeks of age, were obtained from Envigo. They were housed in temperature- and humidity-controlled room with a 12 h light/dark cycle. Mice were offered with 19% protein rodent diet and water ad libitum. To establish the melanoma model, 5 × 10^4^ log-phase B16F10 cells (0.1 ml of cell suspension at 5 × 10^5^/ml) were injected intravenously to each mouse via the tail vein.

The oral treatment with endoxifen (5 mice/group) was initiated one day after inoculation. A 22-gauge metal gavage needle was used for dose administration. The dosing volume was calculated based on individual body weight at 20 ml/kg. The dosing solution was freshly prepared daily and administered to the animals for 20 consecutive days.

On day 21, after dissection of the animals, tumor nodules were only found in the lungs. No metastasis was found in other tissues. The lungs were dissected, weighed and then fixed in 10% neutral-buffered formalin solution. The black tumor nodules lodged in the lungs were counted.

### Statistics

Data are presented as means ± SD. Statistical significance was determined using Student’s *t* test (*p* < 0.05).

## Results

### In vitro anti-melanogenic activity

**Fig. 1:**
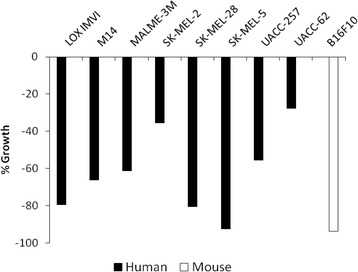
Endoxifen activity in cultured cell lines. Cells were incubated with 10 μM endoxifen for 48 h. Results are shown as a percentage of the cell growth measured for control cells. A 100% growth indicates a comparable growth rate with untreated control cells indicating no activity. A 0% growth indicates a complete growth arrest. A negative value indicates cell death

Treatment with endoxifen at 10 μM for 48 h resulted in significant cell death across all melanoma cell lines tested (Fig. 1). Treatment of B16F10 mouse melanoma and SK-MEL-5 human melanoma cell lines with endoxifen respectively resulted in 93.6 and 92.5% cell death.

### Preclinical safety study in mice

Orally administered endoxifen was well tolerated in Swiss albino mice following 28 days of treatment at a daily dose of up to 8 mg/kg body weight. There were no mortalities, clinical signs of toxicity, or abnormalities observed in gross pathological examinations. Histopathological examinations revealed effects such as mild reduction in the weight of uterus and mild atrophy of myometrial glands of the uterus in female animals.

### In vivo anti-melanogenic activity

The anti-melanoma efficacy of endoxifen was tested in B16F10 melanoma-bearing C57BL/6 mice. The B16F10 cell line was selected for in vivo testing due to its high in vitro sensitivity to endoxifen and its in vivo aggressiveness. For comparison, tamoxifen was also included in the study.

**Fig. 2:**
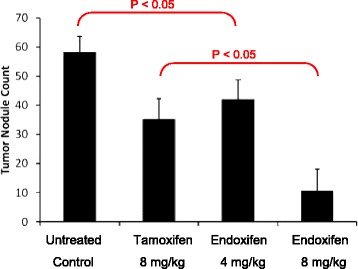
Therapeutic efficacy of endoxifen in the B16F10 melanoma tumor model in mice. Endoxifen or tamoxifen significantly reduced nodule counts when compared to the control group (*p* < 0.05). At an equal dose of 8 mg/kg body weight, endoxifen exhibited significantly stronger activity in reducing melanoma nodule counts than tamoxifen (*p* < 0.05). The data represent means ± SD, (*n* = 5)

As shown in Fig. 2, an average of 58 melanoma nodules developed in the untreated control group during the study. Treatment with tamoxifen at a dose of 8 mg/kg body weight reduced the metastatic nodules to 35, a 39.7% reduction. At same dose level, endoxifen (8 mg/kg) resulted in an 82.7% reduction in nodule count, which represents a significant inhibition in tumor growth.

**Fig. 3:**
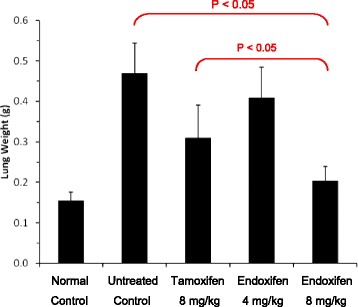
Comparative lung weights of normal, untreated and drug-treated melanoma mice. Treatment with endoxifen or tamoxifen at a dose of 8 mg/kg body weight significantly reduced lung weight of mice with melanoma (*p* < 0.05). The data represent the means ± SD, (*n* = 5)

As an auxiliary therapeutic indicator, the weight of the lungs was also evaluated and compared for normal mice and those with melanoma (Fig. 3). The average weight of the lungs was 0.154 g for normal mice and 0.469 g for untreated melanoma mice on day 21. The increased lung weight was due to the rapid progression and growth of the inoculated melanoma cells. Treatment with tamoxifen at a dose of 8 mg/kg significantly reduced the average lung weight to 0.309 g, a 34% reduction. Treatment with endoxifen at dose levels of 4 mg/kg and 8 mg/kg reduced the lung weight to 0.409 g (13% reduction) and 0.203 g (57% reduction), respectively. Treatment with endoxifen resulted in a dose-dependent growth inhibition of melanoma in this mouse model. At an equal dose, endoxifen is significantly more active than tamoxifen.

## Discussion

In this study, micro-molar concentrations of endoxifen were demonstrated to be highly active to all human and mouse melanoma cell lines. Furthermore, orally administered endoxifen was found to be safe for 28 days of treatment in mice and effective in inhibiting the growth of intravenously inoculated B16F10 cells in C57BL/6 mice (Figs. 2 and 3).

Although the exact mechanism of endoxifen inhibition in melanoma is unclear, it is believed that endoxifen exerts its effect in part via estrogen receptor (ER), in a similar manner to tamoxifen in ER-positive breast cancer cells. At nano-molar concentrations, endoxifen can effectively block ER transcriptional activity, resulting in growth arrest in MCF-7 cells. In fact, the anti-estrogen activity of endoxifen is up to 100 times more potent than that of tamoxifen [10]. However, we observed significant endoxifen anti-melanogenic activity at micro-molar concentrations. At 10 μM, endoxifen resulted in significant cell death in all melanoma cell lines tested over a 48-h period (Fig. 1).

The presence of ER in clinical melanoma biopsies has been confirmed previously [11, 12]. It was found that ER-β, not ER-α, is the predominant subtype in all primary melanoma tumors [13]. Tamoxifen was found to suppress protein kinase C (PKC) and other signaling pathways in mouse melanoma cell line B16BL6 [14]. Interestingly in a recent double blind, active-controlled trial, it was demonstrated that endoxifen possess antimanic efficacy, potentially by inhibiting PKC activity [15].

A recent study demonstrated that endoxifen binds and inhibits PKCβ1 with an IC_50_ of 350 nM [16]. Therefore, endoxifen or tamoxifen may work as ER-α and ER-β modulators and/or by modulating other non-genomic pathways. The activity of endoxifen in melanoma cells is more profound than that in non-melanoma and non-neoplastic cell lines [17].

Orally administered endoxifen is readily bioavailable and rapidly diffuses into tissues in mice [18] and rats [9]. A single 10 mg/kg body weight oral dose yielded a maximum plasma concentration (C_max_) of 34 ng/ml and the endoxifen was found to accumulate in the plasma over a 5-day dose regimen [18]. A 20-day administration schedule would result in a significant drug accumulation in the plasma and other tissues, like the lungs, and in lung-associated melanoma lesions.

It is interesting to note that the therapeutic dose level of 8 mg/kg body weight in our study is equivalent to a human dose of 24 mg/m^2^ body surface area or 40 mg/day for an adult. This dose level is within the range (20–160 mg) of a recent phase I clinical study in patients with endocrine-refractory, metastatic breast cancer where endoxifen demonstrated acceptable toxicity and promising antitumor activity [19]. Thus, with a favorable toxicity and safety profile, endoxifen may be a new promising therapeutic agent worthy of further evaluation.

## Abbreviations

C_max_: maximum plasma concentration
CYP2D6: cytochrome P450 2D6
DMEM: Dulbecco’s modified Eagle’s medium
ER: estrogen receptor
FBS: fetal bovine serum
PKC: protein kinase C
SRB: sulforhodamine B
WST-1: (4-[3–4-iodophenyl]-2-(4-nitrophenyl)-2*H*-5-tetrazolio)-1,3-benzene disulfonate)
